# Hoffa’s fat pad thickness: a measurement method with sagittal MRI sequences

**DOI:** 10.1007/s11547-021-01345-9

**Published:** 2021-03-27

**Authors:** Giovanni Ricatti, Nicola Veronese, Ilaria Gangai, Mariateresa Paparella, Valentina Testini, Giuseppe Guglielmi

**Affiliations:** 1 Department of Clinical and Experimental Medicine, School of Medicine, Foggia University, Viale L. Pinto, 1, 71121 Foggia, Italy; 2 Department of Internal Medicine and Geriatrics, Geriatric Unit, University of Palermo, Palermo, Italy; 3 Department of Radiology, “Dimiccoli” Hospital, Barletta, Italy; 4 Department of Radiology, Hospital “Casa Sollievo Della Sofferenza”, San Giovanni Rotondo, Foggia, Italy

**Keywords:** Hoffa’s fat pad, Infrapatellar fat pad, Knee joint, OAI, MRI

## Abstract

**Background:**

Hoffa’s fat pad is a structure located within the fibrous joint capsule of the knee joint, but outside the synovial cavity. It plays an important biomechanical and metabolic role in knee joint, reducing the impact of forces generated by loading and producing cytokines. Changes in its size can induce modifications in the knee homeostasis. However, a great variability exists regarding its measurements. This work aims to evaluate the reliability of a measurement method of Hoffa’s fat pad dimensions through MRI.

**Methods:**

3T sagittal IW 2D TSE fat-suppressed MRI sequences, taken from the OAI (Osteoarthritis initiative) database, of 191 male and female patients, aged between 40 and 80 years, were analysed; a manual measurement of the thickness of Hoffa’s fat pad of each subject was then performed by two different readers. The interobserver reliability and intraobserver reliability of the measurements were described by coefficient of variation (CV), Pearson correlation and Bland–Altman plots.

**Results:**

All statistical analyses have shown that not significant intra- or interobservers differences were evident (intraobserver CV % for the first observer was 2.17% for the right knee and 2.24% for the left knee, while for the second observer 2.31% for the right knee and 2.24% for the left knee; linear correlation was for the first observer *r* = 0.96 for the right knee and *r* = 0.96 for the left knee, while for the second observer *r* = 0.97 for the right knee and *r* = 0.96 for the left knee; in addition, the interobserver CV % was 1.25% for the right knee and 1.21% for the left knee and a high interobserver linear correlation was found: *r* = 0.97 for the right knee and *r* = 0.96 for the left knee).

All results suggest that this manual measurement method of Hoffa’s fat pad thickness can be performed with satisfactory intra- and interobserver reliability.

**Conclusions:**

Hoffa’s fat pad thickness can be measured, using sagittal MRI images, with this manual method that represents, for his high reliability, an effective means for the study of this anatomical structure.

## Introduction

Hoffa's fat pad, also known as infrapatellar fat pad (IPFP), is an intracapsular but extra-synovial structure of the anterior knee joint [1, 2]. It is limited by the patellar tendon anteriorly and the synovial-lined knee joint posteriorly. Superiorly, it attaches to the inferior surface of the patella, while superoposteriorly Hoffa's fat pad neighbours the cartilage of the femoral trochlea. Inferiorly instead IPFP reaches the periosteum of the tibia [3, 4].

It is structurally composed of adipose tissue similar to subcutaneous fat [5] and plays an important biomechanical role to reduce the impact of forces generated by loading in the knee joint [6, 7]. Moreover, IPFP has a great relevance in knee joint metabolism and a central role in joint inflammation [8–10], because of the production of cytokines such as interleukin (IL)-6, tumour necrosis factor (TNF)-a and adipokines, such as leptin [11–13].

High levels of leptin have been found in the synovial fluid of osteoarthritis patients. There is also a correlation between synovial fluid leptin concentration with disease severity and a significantly higher leptin expression in the IPFP and synovial tissues of OA patients [14]. Hence, it is possible to assume that variations in IPFP thickness and morphology, causing a change in the amount of adipose tissue cells, may induce alterations in inflammatory cytokines levels and thus may cause articular tissue pathology, as observed in knee osteoarthritis.

These elements acquire great relevance if we consider that knee osteoarthritis represents the most common form of arthritis and is a frequent cause of chronic disability. Moreover, knee OA is responsible for a huge number of quality-adjusted life-years lost in older men and women.

In the field of diagnostic imaging, Conventional Radiology has long been considered the reference standard, and multiple ways to define radiographic disease of OA have been devised. The most common method for radiographic definition is the Kellgren–Lawrence (K/L) radiographic grading scheme and atlas. This overall joint scoring system grades Osteoarthritis in five levels from 0 to 4, defining OA by the presence of a definite osteophyte (Grade ≥ 2), and more severe grades by the successive appearance of joint space narrowing, sclerosis, cysts, and deformity [15]. Other radiographic metrics including semiquantitative examination of radiographic features, such as osteophytes and joint space narrowing, or the direct measurement of the interbone distance as an indicator of the joint space width in the knees are often used to investigate progression of the disease [16, 17].

However, in the last years, MRI [18, 19] has become the gold standard for the study of knee joint, including Hoffa’s fat pad [20, 21]. In particular, magnetic resonance imaging allows the assessment of inflammatory disease manifestations. This is usually performed, for the study of IPFP, using surrogate signal changes in Hoffa’s fat pad on nonenhanced MRI or by direct assessment of the synovium on contrast-enhanced scans [22–24].

Other MRI applications in the field of OA research include dynamic contrast-enhanced MRI, which allows the evaluation of inflammatory synovial activity by assessing the degree of perfusion of the synovial tissue [25].

In addition, most recently, the volume of Hoffa’s fat pad has become a subject of interest and methods of segmentation and measurement of Hoffa's fat pad have been proposed, both manual and automatic through the use of specific software. [26].

The purpose of this study was to demonstrate the validity of a manual method of measuring the thickness of Hoffa’s fat pad, highlighting both intra- and interobserver reliability, in consideration of the described importance of the modifications of this structure and their connections with osteoarthritis.

## Materials and methods

### Data source and subjects

**Fig. 1:**
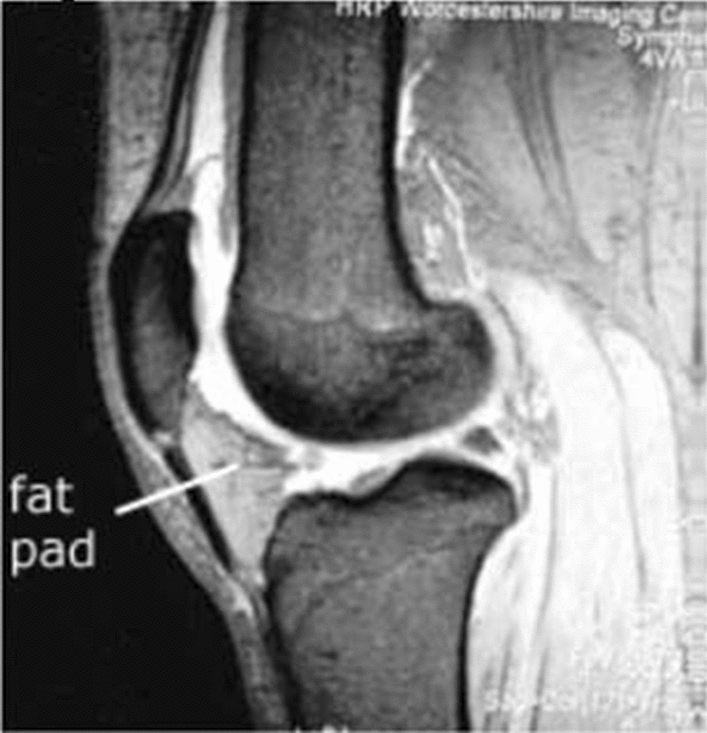
Fat pad in knee joint. 3T sagittal intermediate-weighted (IW) 2D TSE Fat-suppressed MRI sequence

The data used in this study were obtained from the Osteoarthritis initiative (OAI) database, a multicentre, longitudinal, prospective observational study of knee osteoarthritis [27, 28]. The OAI participants were recruited, between February 2004 and May 2006, at four American Universities (the University of Maryland School of Medicine and Johns Hopkins University School of Medicine, the Ohio State University, the University of Pittsburgh and the Memorial Hospital of Rhode Island). The study was approved by the local ethics committees and by the Committee on Human Research of the Institutional Review Board for the University of California, San Francisco, and informed consent was obtained from all participants (Fig. 1).

### Image acquisition and analysis

For the analysis of the IPFP, a sagittal intermediate-weighted fat-suppressed turbo spin-echo sequence, IW TSE, was used (time of repetition = 3200 ms, time of echo = 30 ms, slice thickness 3.0 mm; in plane resolution 0.36 mm × 0.36 mm). These images were acquired using a 3T Magnetom Trio magnet (Siemens Healthcare Erlangen, Germany) and a quadrature knee coil [29, 30].

Brightness, intensity, contrast, and grey value limits were adjusted manually in each image to warrant optimal contrast between the IPFP and surrounding tissue.

**Fig. 2:**
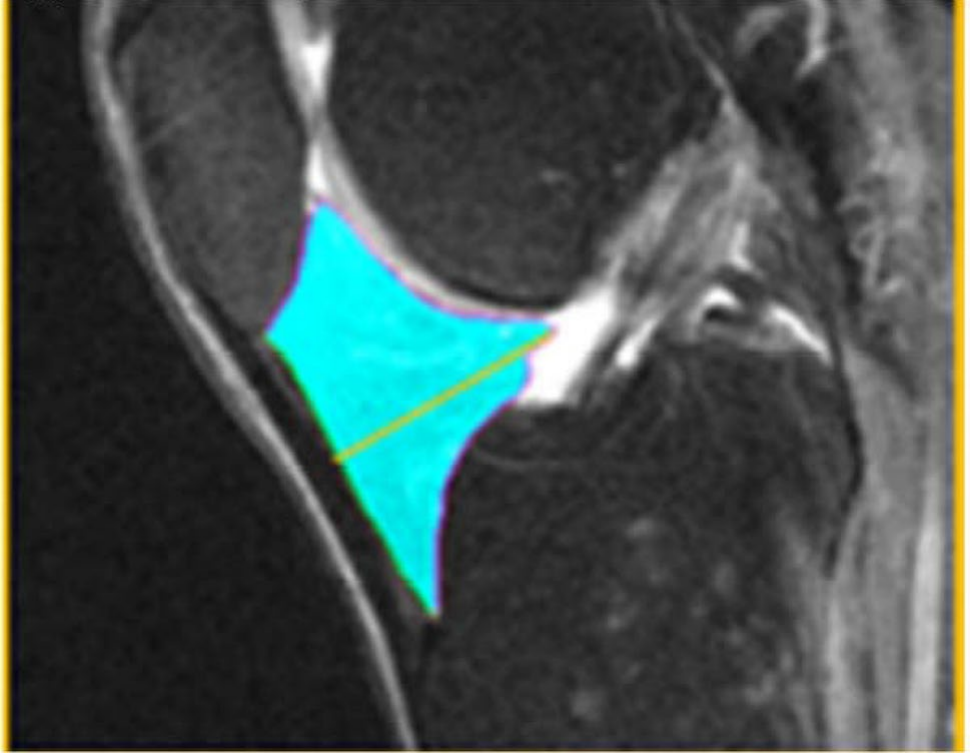
Fat pad maximal sagittal thickness. Manual measurement on 3T sagittal IW 2D TSE Fat-suppressed MRI sequence

In our study, for both the right knee and the left knee, the maximal sagittal thickness (depth) of IPFP, from the anterior to posterior surface, was manually measured for each patient, drawing a line perpendicular to the patellar tendon (Fig. 2).

All these measurements were performed by two different blinded observers (G.R, I.G.), both Radiology residents with 3 years of experience; the intraobserver reliability was determined from baseline and after 1-month review images; in addition, all measurements were quality controlled by an expert radiologist with experience in image analysis of musculoskeletal tissues [31].

### Statistics

All statistical analyses were performed using SPSS 21.0. After checking the normality of the distribution of the continuous variables, quantitative measurements of IPFP thickness were reported as means and standard deviations (SDs), while categorical variables as frequencies and percentages.

We assessed the reliability of the measurements through different methods.

First, the interobserver reliability and intraobserver reliability of the measurements were described by a coefficient of variation (CV) in percent (%) (larger values indicate greater variability and consequently a lower degree of reliability) [32]. To evaluate whether there were significant differences between baseline and 1-month review of the same investigator (intraobserver variability), a paired *t* test was applied; the mean differences between two different readers (interobserver variability) were analysed through an independent group *t*-test.

Second, the Pearson correlation was computed to evaluate the strength of the association between two different measures and the results were reported as r, with the correspondent *p*-value.

Finally, we used the Bland–Altman plots (BAPs), a method to quantify agreement between two quantitative measurements by studying the mean difference and constructing limits of agreement. It is a simple way to evaluate, with a graphic approach, a bias between the mean differences, and to estimate an agreement interval, within which 95% of the differences of the second method, compared to the first one, fall. Data can be analysed both as unit differences plot and as percentage differences plot.

Since no significant differences for intra- or interobservers were evident, we finally reported the results as means of the different measures made by the two investigators.

## Results

The OAI study initially included 4096 participants. For 796 patients, MRI of the knees was available. For 191 of them, the image quality of sagittal MRI sequences allowed an adequate manual measurement of Hoffa’s fat pad thickness, so they were included in our work. Hoffa’s fat pad pathology was not an exclusion criteria.

### Intraobserver evidence

The intraobserver CV % values obtained for IFPF thickness showed a high degree of reliability for both the first observer (2.17% for the right knee and 2.24% for the left knee) and the second observer (2.31% for the right knee and 2.24% for the left knee) (Table 1).

**Table 1:** Coefficient of variation

Intraobserver CV					
		CV right observer 1	CV left observer 1	CV right observer 2	CV left observer 2
N	Valid	191	191	191	191
	Missing	21	21	21	21
CV (%)		2.1757	2.2448	2.3184	2.2448
Standard deviation		2.31004	1.92711	1.50358	2.46667
Minimum		0.00	0.00	0.00	0.00
Maximum		23.57	15.71	6.73	28.28

Interobserver CV			
		CV left observer 1	CV right observer 2
*N*	Valid	191	191
	Missing	21	21
CV		1.2515	1.2087
Standard deviation		1.37297	1.50436
Minimum		0.00	0.00
Maximum		7.79	10.48

Moreover, no statistically significant differences between baseline and 1-month review were found for all the observers (first observer: *p* = 0.14 for the right knee and *p* = 0.15 for the left knee; second observer: *p* = 0.25 for the right knee and *p* = 0.26 for the left knee).

In addition, a high linear correlation between baseline and 1-month review was found for both the first and the second observer (first observer: *r* = 0.96 for the right knee and *r* = 0.96 for the left knee; second observer: *r* = 0.97 for the right knee and *r* = 0.96 for the left knee) (Table 2).

**Table 2:** Pearson correlation

Intraobserver correlations					
		Right knee first observer measurement 2	Left knee first observer measurement 2	Right knee second observer measurement 2	Left knee second observer measurement 2
Right knee first observer measurement 1	Pearson correlation	0.962**	0.775**	0.971**	0.783**
	Sig. (2-code)	0.000	0.000	0.000	0.000
	*N*	191	191	191	191
	Sig. (2-code)		0.000	0.000	0.000
	*N*	191	191	191	191
Left knee first observer measurement 1	Pearson correlation	0.782**	0.961**	0.802**	0.958**
	Sig. (2-code)	0.000	0.000	0.000	0.000
	*N*	191	191	191	191
	Sig. (2-code)	0.000		0.000	0.000
	*N*	191	191	191	191
Right knee second observer measurement 1	Pearson correlation	0.947**	0.798**	0.969**	0.811**
	Sig. (2-code)	0.000	0.000	0.000	0.000
	*N*	191	191	191	191
	Sig. (2-code)	0.000	0.000		0.000
	*N*	191	191	191	191
Left knee second observer measurement 1	Pearson correlation	0.790**	0.954**	0.815**	0.964**
	Sig. (2-code)	0.000	0.000	0.000	0.000
	*N*	191	191	191	191
	Sig. (2-code)	0.000	0.000	0.000	
	*N*	191	191	191	191

Interobserver correlations					
		Right knee first observer	Left knee first observer	Right knee second observer	Left knee second observer
Right knee first observer	Pearson correlation	1	0.807**	0.969**	0.810**
	Sig. (2-code)		0.000	0.000	0.000
	*N*	191	191	191	191
Left knee first observer	Pearson correlation	0.807**	1	0.819**	0.973**
	Sig. (2-code)	0.000		0.000	0.000
	*N*	191	191	191	191
Right knee second observer	Pearson correlation	0.969**	0.819**	1	0.835**
	Sig. (2-code)	0.000	0.000		0.000
	*N*	191	191	191	191
Left knee second observer	Pearson correlation	0.810**	0.973**	0.835**	1
	Sig. (2-code)	0.000	0.000	0.000	
	*N*	191	191	191	191

**The correlation is statistically significant at the 0.001 level (2-code)

**Fig. 3:**
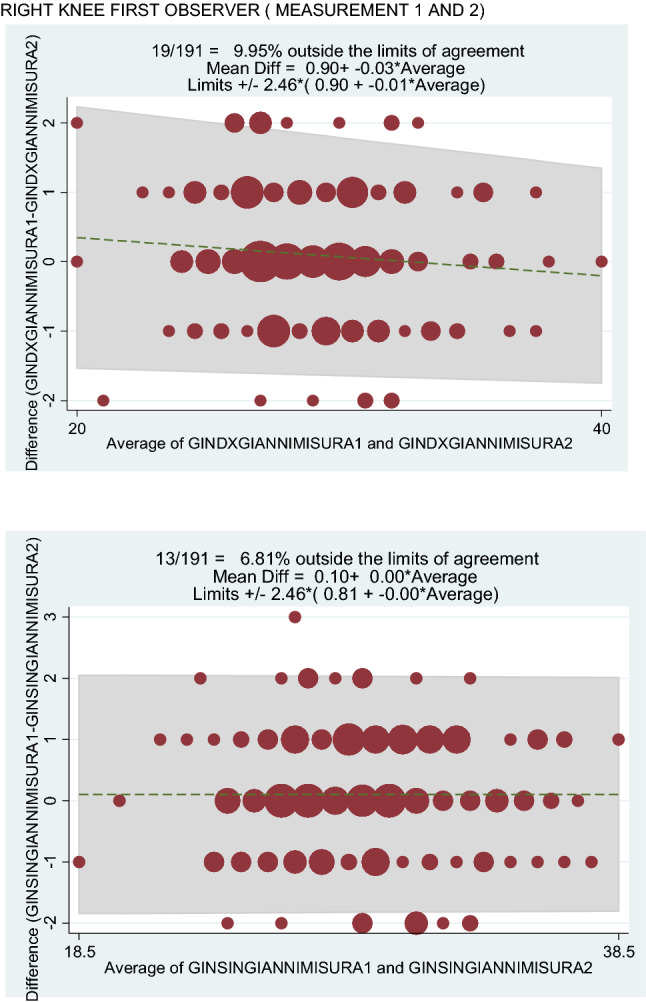
Right knee first observer (measurement 1 and 2). Left knee first observer (measurements 1 and 2)

**Fig. 4:**
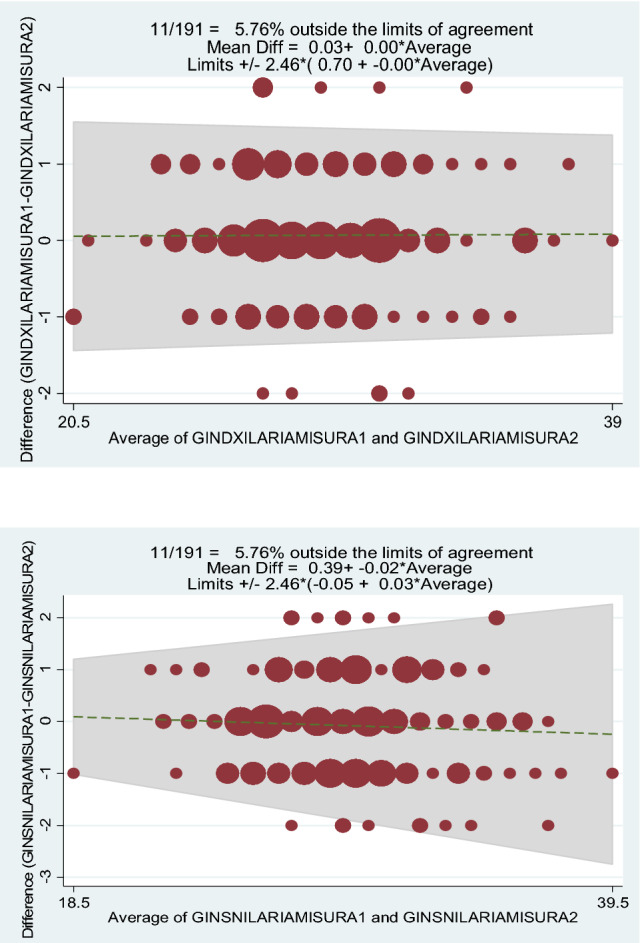
Right knee second observer (measurement 1 and 2). Left knee second observer (measurements 1 and 2)

The Bland–Altman method also showed the intraobserver reliability of the measurement method, because only a few values obtained were outside the limits of agreement (first observer: 9.9% for the right knee and 6.8% for the left knee (Fig. 3); second observer: 5.8% for both knees) (Fig. 4).

### Interobserver evidence

The statistical analyses also showed the high interobserver reliability of the measurement method.

In fact, the interobserver CV % values for Hoffa’s fat pad thickness were found really low for both the right and the left knee (1.25% for the right knee and 1.21% for the left knee) (Table 1).

No statistically significant differences between observers were found for both knees (*p* = 0.25 for the right knee and *p* = 0.94 for the left knee).

In addition, a high linear correlation between the values obtained by the two observers was found (*r* = 0.97 for the right knee and *r* = 0.96 for the left knee) (Table 2).

**Fig. 5:**
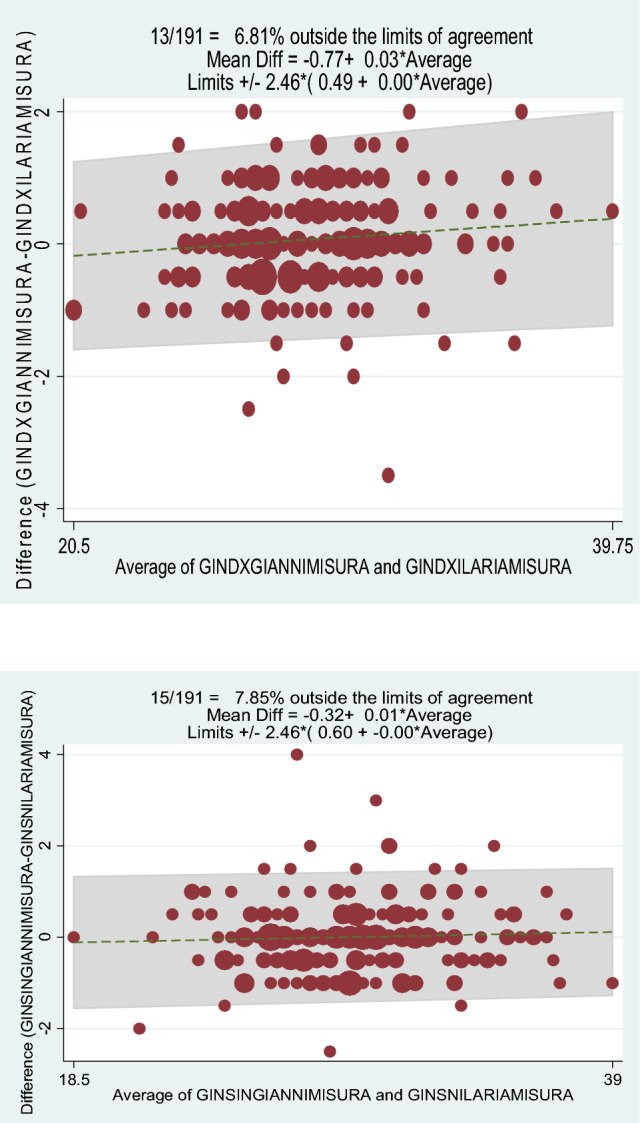
Right knee interobserver. Left knee interobserver

The Bland–Altman method also highlighted the interobserver reliability of the measurement method, revealed by the fact that only a few values obtained were outside the limits of agreement (6.8% for the right knee and 7.8% for the left knee) (Fig. 5).

## Discussion

In this work, we reported the reliability and the accuracy of the measurement of the Hoffa’s fat pad, by two independent investigators, made using a 3T MRI.

It is particularly important to estimate dimensions of Hoffa’s fat pad because variations in its volume and thickness can result in degenerative changes of the knee joint, as seen for example in Osteoarthritis, reducing physical performance and consequently abilities to perform well in activities of daily living, becoming a cause of chronic disability [33].

Recent scientific works have also highlighted the existence of a relationship between the volume of the Hoffa’s fat pad and obesity.

In fact, an increase in the thickness and in the volume of IPFP was observed in subjects with a high body mass index, suggesting a potential endocrine link between obesity and Osteoarthritis, with more intra-articular adipose tissue potentially releasing greater amounts of adipokines, inducing inflammation.

Moreover, it has recently been reported in some works that IPFP volume may be responsive to exercise and diet as treatment of knee OA, maybe because the combination of exercise and diet can reduce IPFP volume, providing clinical improvement in knee OA [34, 35].

In addition, the possibility of exploring sex differences in Hoffa’s fat pad volume and thickness and of determining the thickness of IPFP in various age groups can be of great interest in order to highlight any changes related, for example, to sex and aging [36].

It is therefore clear that the determination of the size of the Hoffa’s fat pad, through a standard and reproducible method, may be of great importance, especially in all those areas of Medicine that study metabolic and degenerative pathologies.

In this sense, the role of the radiologist is fundamental, who is responsible for adequately determining dimensions of the IPFP.

There are many different morphological measurements of Hoffa's fat pad that can be considered: volume, anterior and posterior surface area, maximum sagittal thickness (depth), maximum sagittal area and central slice area; these are often obtained with custom segmentation software.

In our work, we have proposed a manual measurement method of Hoffa’s fat pad maximum thickness, using sagittal 3T MRI images.

An important element of this method is that the values are obtained without the use of IPFP segmentation software and measurements can be performed with relative speed, even by young trained researchers, as seen in our experience, allowing application even in smaller radiological departments [37].

A comparison between manual measurement method and automated method was not performed because automated software was not available in our department, when writing the article.

However, it could be useful, in future works, to realize a comparison between different measurement methods, including the one proposed in the study.

A limitation of this work can be represented by the fact that interobserver reliability has been tested only between two observers; however, in consideration of the fact that all statistical analyses did not show statistically significant differences between the two observers, it is reasonable to believe that this measurement method can also be reproduced by other operators.

## Conclusion

The study of Hoffa’s fat pad has a growing importance to understand pathogenesis and therapy of many metabolic and degenerative diseases.

Hoffa’s fat pad thickness can be measured, using sagittal MRI images, with this manual method that represents, for his high inter- and intraobserver reliability, an effective means for the study of this anatomical structure.

Finally, the possibility of exploring sex differences in Hoffa’s fat pad thickness and of determining the thickness of IPFP in various age groups, using this measurement method, can be of great interest in order to highlight any changes related, for example, to sex and aging.
